# The sRNA Regulated Protein DdbA Is Involved in Development and Maintenance of the Chlamydia trachomatis EB Cell Form

**DOI:** 10.3389/fcimb.2021.692224

**Published:** 2021-07-23

**Authors:** Nicole A. Grieshaber, Justin Runac, Sierra Turner, Marissa Dean, Cody Appa, Anders Omsland, Scott S. Grieshaber

**Affiliations:** ^1^ Department of Biological Sciences, University of Idaho, Moscow, ID, United States; ^2^ College of Dentistry, University of Florida, Gainesville, FL, United States; ^3^ Paul G. Allen School for Global Animal Health, College of Veterinary Medicine, Washington State University, Pullman, WA, United States

**Keywords:** Chlamydia, bacterial cell development, bacterial replication, elementary bodies, reticulate body

## Abstract

The chlamydial small non coding RNA, IhtA, regulates the expression of both HctA and DdbA, the uncharacterized product of the C. trachomatis L2 CTL0322 gene. HctA is a small, highly basic, DNA binding protein that is expressed late in development and mediates the condensation of the genome during RB to EB differentiation. DdbA is conserved throughout the chlamydial lineage, and is predicted to express a small, basic, cytoplasmic protein. As it is common for sRNAs to regulate multiple mRNAs within the same physiological pathway, we hypothesize that DdbA, like HctA, is involved in RB to EB differentiation. Here, we show that DdbA is a DNA binding protein, however unlike HctA, DdbA does not contribute to genome condensation but instead likely has nuclease activity. Using a DdbA temperature sensitive mutant, we show that DdbAts creates inclusions indistinguishable from WT L2 in size and that early RB replication is likewise similar at the nonpermissive temperature. However, the number of DdbAts infectious progeny is dramatically lower than WT L2 overall, although production of EBs is initiated at a similar time. The expression of a late gene reporter construct followed live at 40°C indicates that late gene expression is severely compromised in the DdbAts strain. Viability assays, both in host cells and in axenic media indicate that the DdbAts strain is defective in the maintenance of EB infectivity. Additionally, using Whole Genome Sequencing we demonstrate that chromosome condensation is temporally separated from DNA replication during the RB to EB transition. Although DdbA does not appear to be directly involved in this process, our data suggest it is a DNA binding protein that is important in the production and maintenance of infectivity of the EB, perhaps by contributing to the remodeling of the EB chromosome.

## Introduction

*Chlamydiae* are bacterial pathogens that are responsible for a wide range of diseases in both animal and human hosts. *Chlamydia trachomatis* (*Ctr*), a human pathogen, is comprised of over 15 distinct serovars causing both trachoma, the leading cause of preventable blindness, and sexually acquired infections of the urogenital tract. According to the CDC, *Ctr* is the most frequently reported sexually transmitted infection in the United States, costing the American healthcare system nearly $2.4 billion annually (Datta et al., 2012; Edusei et al., 2013). These infections are widespread among all age groups and ethnic demographics, infecting ~3% of the human population worldwide (Torrone et al., 2014; Torrone et al., 2014). In women, untreated genital infections can result in devastating consequences such as pelvic inflammatory disease, ectopic pregnancy, and infertility (Miller et al., 2004; Ohman et al., 2009). Every year, there are over 4 million new cases of Chlamydia in the United States (Miller et al., 2004; Datta et al., 2012) and an estimated 92 million cases worldwide (World Health Organization, 2001).

Chlamydial disease is entirely dependent on the establishment and maintenance of the unique intracellular niche where the bacteria replicate and carry out a biphasic developmental cycle. *Chlamydiae* are exquisitely adapted to life inside cells and cycle through two unique developmental forms: the elementary body (EB) and the reticulate body (RB). Following invasion of a host cell *via* pathogen mediated endocytosis, the EB differentiates into the RB cell type. After several rounds of replication, a subset of RBs re-differentiate back into the EB form allowing another round of infection following release from the host cell (Abdelrahman and Belland, 2005; Chiarelli et al., 2020). The molecular events that control differentiation between the two cell forms are poorly understood. We previously described a small regulating non coding RNA (sRNA), IhtA, that regulates the expression of the chlamydial HctA protein (Grieshaber et al., 2006). HctA is a small, highly basic protein expressed late in development that interacts with DNA and mediates the condensation of the chlamydial genome during RB to EB differentiation (Hackstadt et al., 1991; Tao et al., 1991). IhtA interacts with the *hctA* mRNA *via* a 7nt binding region which includes a G/C clamp surrounding the AUG start site to shut down translation of the message (Grieshaber et al., 2012). Characterization of the IhtA binding site within the HctA transcript led to the identification of a second protein whose expression was regulated by IhtA, the hypothetical protein CTL0322, which we have termed developmental DNA binding protein (DdbA) (Grieshaber et al., 2012). As it is common for sRNAs to regulate multiple mRNAs within the same physiological pathway, we hypothesized that DdbA, like HctA, is involved in RB to EB differentiation. Indeed, a DdbA temperature sensitive mutant has been shown to be defective at the production of infectious progeny at the non-permissive temperature between 18 and 24 h which corresponds to the beginning of RB to EB differentiation in *Ctr* (Brothwell et al., 2014).

In this study we show that the product of the ddbA gene is a DNA binding protein with potential nuclease activity. We also demonstrate that DdbA is involved in the development/maintenance of the EB as the DdbAts mutant appears to create normal inclusions and the early RBs replicate at a similar rate as the wild type but EB production/survival is dramatically reduced. Intriguingly, DdbA is regulated by the same sRNA as HctA suggesting that IhtA plays an important role in EB biology.

## Material and Methods

### Cell Culture

Cell lines were obtained from American Type Culture Collection. HeLa 229 cells (CCL-2.1), and Cos-7 cells (CRL-1651) were grown in RPMI-1640, supplemented with 10% FBS and 10 μg/mL gentamicin (Cellgro). *Chlamydia trachomatis* serovar L2 (LGV Bu434) was grown in Cos-7 cells. Elementary Bodies (EBs) and Reticulate bodies (RBs) were purified by density gradient (DG) centrifugation essentially as described (Howard et al., 1974) following 17 or 43-45 h of infection, respectively. EBs were stored at -80°C in Sucrose Phosphate Glutamate (SPG) buffer (10 mM sodium phosphate [8mM K2HPO4, 2mM KH2PO4], 220 mM sucrose, 0.50 mM l-glutamic acid, pH 7.4) until use.

### Vector Construction

Invitrogen Gateway Technology was used for expression of the nucleic acid-binding proteins DdbA and HctA. Each gene ORF was cloned into pEXP1-DEST to express N-terminal 6xHis tagged proteins and into pET104 Bioease to express N-terminal biotin tagged proteins. Each gene was also subcloned into the destination vector pcDNATM6.2/EmGFP for expression of chlamydial proteins in eukaryotic cells. The N-EmGFP-DEST vector was used for eukaryotic expression of N-terminal GFP-tagged proteins in mammalian cells. The leading CACCATG sequence was used as the Kozak translation initiation sequence for all C-terminally tagged proteins in mammalian cells (Xia, 2007).

A L2 genomic library was constructed using Novagen’s pET-41a (+) vector. A RsaI digestion of Ctr L2 genomic DNA and electrophoresis were performed to purify 500 bp fragments. These fragments were electro-eluted and ligated into pET-41a(+) and include an N-terminal GST tag and a C-terminal 6xHis tag when in proper frame.

All *Ctr* expression constructs used p2TK2SW2 (Wang et al., 2011) as the backbone and protein expression was regulated by an E-riboswitch (Topp and Gallivan, 2007). E-clover-6xHis and E-clover-3xFlag fragments were ordered as a geneblock from Integrated DNA technologies (IDT) and inserted between the T5 promoter and IncD terminator of p2TK2SW2. The Euo, HctA, Scc1 and DdbA ORFs were amplified from *Ctr* L2 and each fragment was used to replace Clover, generating E-Euo-6xHis and E-DdbA-6xHis expression constructs and E-DdbA-3xFlag, E-HctA-3xFlag and E-Scc1-3xFlag constructs.

### Chlamydial Transformation and Isolation

Transformation of *Ctr* L2 was performed essentially as previously described (Wang et al., 2011). Briefly, 1x10^8^ EBs + 2µg DNA/well were used to infect a 6 well plate. Transformants were selected with 1U/ml Penicillin G or 500µg/ml Spectinomycin as appropriate for plasmid. Each new strain was clonally isolated *via* successive rounds of inclusion isolation (MOI, <1) using a micromanipulator. Clonality of each strain was confirmed by isolating the plasmid, transforming into *E. coli* and sequencing six transformants.

### Microscopy

Fluorescence images were acquired using a Nikon spinning disk confocal system with a 60x oil-immersion objective, equipped with an Andor Ixon EMCCD camera, under the control of the Nikon elements software. Images were processed using the image analysis software ImageJ (http://rsb.info.nih.gov/ij/). Representative confocal micrographs displayed in the figures are maximal intensity projections of the 3D data sets, unless otherwise noted.

Live cell imaging of inclusions expressing the fluorescent protein Clover (Lam et al., 2012) under the control of the *hctA* promoter (Grieshaber et al., 2012; Chiarelli et al., 2020) was achieved using an automated Nikon epifluorescent microscope equipped with an Okolab (http://www.oko-lab.com/live-cell-imaging) temperature controlled stage and an Andor Zyla sCMOS camera (http://www.andor.com). Images were taken every fifteen minutes for 48 hours. Multiple fields of view of multiple wells of a glass bottom 24 well plate were imaged. The fluorescence intensity of each inclusion over time was tracked using the ImageJ plugin Trakmate (Tinevez et al., 2016) and the results were averaged and plotted using python and matplotlib.

### Transfection

All transfections of eukaryotic cells were performed with Invitrogen Lipofectamine 2000 Transfection Reagent. HeLa 229 cells were grown to 70% confluence and transfected with pcDNATM6.2/N-EmGFP-DEST with each of the genes of interest cloned into that destination vector. At 24 hours post-transfection, the HeLa cells were prepared for fixation and staining.

### Fluorescence Staining

#### Mammalian Ectopic Expression

The HeLa cells used in the ectopic chlamydial protein expression studies were fixed for 10 minutes with methanol 24 hours post-transfection. The cells were washed 2 times with PBS. Monoclonal Anti-β-tubulin I (Sigma-Aldrich^®^) was used to show cell shape and the far-red fluorescent DNA dye Draq5 (Thermo Scientific^®^) was used to stain the nucleus. Coverslips were mounted on a microscope slide with a MOWIOL^®^ mounting solution (100 mg/mL MOWIOL^®^ 4-88, 25% glycerol, 0.1 M Tris pH 8.5).

#### Ectopic Chlamydial Expression

Cos7 cells on coverslips were infected with *Ctr*-E-DdbA3xFlag, *Ctr*-E-HctA3xFlag, *Ctr*-E-Scc13xFlag strains and induced at 16 hpi with 0.5mM theophylline (Acros Organics, Thermo Scientific™). Samples were fixed with 4% buffered paraformaldehyde at 24 hpi and stained with an anti-Flag antibody (1:500) to visualize expressing *Chlamydia.* DAPI was used to visualize DNA. Coverslips were mounted on a microscope slide with a MOWIOL^®^ mounting solution (100 mg/mL MOWIOL^®^ 4-88, 25% glycerol, 0.1 M Tris pH 8.5).

#### Ctr ddbAts Confocal Microscopy

Cos7 cells on coverslips were infected with wt (G5 isolate) *Ctr* or *ddbAts* mutant *Ctr* for 18 or 36 hours at either 40°C or 37°C before fixation with 4% buffered paraformaldehyde. After fixation the cells were stained with an anti MOMP primary antibody (Fishersci) followed by an anti-mouse Alexa-488 (Fishersci) secondary antibody. DAPI was used to visualize the nuclei of the host cells. Coverslips were mounted on a microscope slide with a MOWIOL^®^ mounting solution (100 mg/mL MOWIOL^®^ 4-88, 25% glycerol, 0.1 M Tris pH 8.5).

### Replating Assay

Chlamydia were isolated by scraping the infected monolayer into media and pelleting at 17200 rcfs. The EB pellets were resuspended in RPMI *via* sonication and seeded onto fresh monolayers in a 96-well microplate in a 2-fold dilution series. Infected plates were incubated for 24 hours prior to fixation with methanol and stained with DAPI and an anti-*Ctr* antibody (Fishersci). The DAPI stain was used for automated microscope focus and visualization of host-cell nuclei and the anti-*Ctr* antibody for visualization of EBs and inclusion counts. Inclusions were imaged using a Nikon Eclipse TE300 inverted microscope utilizing a scopeLED lamp at 470nm and 390nm, and BrightLine band pass emissions filters at 514/30nm and 434/17nm. Image acquisition was performed using an Andor Zyla sCMOS in conjunction with μManager software. Images were analyzed using ImageJ software and custom scripts.

### Recombinant Protein Purification and Analysis

The bacterial expression vector pEXP1 (InvitrogenTM) was used to express N-terminal his-tagged proteins and proteins were expressed using BL21-AI E. coli cells (Invitrogen) Expression of recombinant proteins was induced with 0.4% L-arabinose and 2 mM IPTG. Cells were induced for 4 hours at 370°C Cells were lysed using a French Press. Lysates were centrifuged at 16,000 x g for 10 minutes to pellet insoluble material. HisPurTM Ni-NTA Resin (Thermo Scientific™) was used to purify the his-tagged proteins. Upon completion of the elution step, the eluted proteins were applied to Amicon^®^ ltracel ^®^ Centrifugal Filters to concentrate the purified protein and remove excess imidazole.

To purify 6xHis tagged proteins from Ctr, a fresh monolayer of Cos-7 cells was infected with each Ctr strain at an MOI of 10 and the his-tagged protein was induced at 16 hpi with 0.5mM theophylline (Acros Organics, Thermo Scientific™) for 6h. Cells were scraped into PBS + protease inhibitors on ice and sonicated in 3 bursts for 5 second/burst followed by a 1 minute rest. This sequence was repeated 10 times. Lysates were cleared and the proteins purified as described above.

To analyze purity and size, protein lysates were separated on 12% SDS-PAGE gels and stained directly with either Coomassie Brilliant Blue G-250 Dye (Thermo Scientific™) or SYPRO Orange Protein Gel Stain (Invitrogen™) as per manufacturer instructions, or were transferred to a Nitrocellulose Membrane for western analysis. The membrane was blocked with PBS + 0.1% Tween 20 (PBS-T) and 5% Bovine Serum Albumin (BSA) overnight at 4°C. Membranes were probed with primary antibody anti-6xHis Tag (Clone: HIS.H8, eBioscience™) overnight at 4°C followed by Goat-anti Mouse IgG-HRP secondary antibody (Invitrogen™) at room temperature for 2 hours. The membrane was developed with the Supersignal West Dura luminol and peroxide solution (Thermo Scientific™) and imaged using an Amersham Imager 600.

### Biolayer Interferometry (BLI)

Customized ssDNA oligonucleotides to generate RNA and dsDNA were ordered from Integrated DNA Technologies. Each sequence was 100 nucleotides in length and contained a T7 promoter at the 5’ end and a T7 terminator at the 3’ end to allow for both PCR and T7 polymerase *in vitro* transcription. The remaining sequences were random nucleotides that were 80%, 60%, or 40% G-C ssDNA.

dsDNA: REDTaq DNA Polymerase was incubated with customized ssDNA, T7 terminator complement primers, and dNTPs in the supplied polymerase buffer. After heat activation of the polymerase, primers were annealed at 55°C. Extension was done at 72°C for 30 minutes. The annealing and extension cycles were repeated 4 times. The synthesized dsDNA was purified using QIAGEN’s QIAquick PCR purification kit to remove all unincorporated primers and dNTPs.

RNA: The MEGAshortscript Kit (Thermo Scientific™) was used to synthesize 100 base pair transcripts from the customized ssDNA oligonucleotides as per manufacturer instructions. Bio-Rad™ Micro Bio-Spin P-6 Gel Chromatography Columns were used to remove unincorporated nucleotides.

BLI: Biolayer interferometry studies of protein-nucleic acid were performed using the Octet QKe (fortéBIO, Menlo Park, CA). Ni-NTA Dip and Read Sensors were equilibrated in binding buffer (0.1 M phosphate, 0.15 M sodium chloride, pH 7.2) for 15 minutes prior to starting the experiment. His-tagged proteins were incubated in the Octet platform with the sensors for 15 minutes. The sensors bound with protein were then incubated with binding buffer to remove unbound protein and establish a baseline for kinetics measurement. The sensors were then incubated for two minutes in either GC-rich or AT/AU-rich nucleic acid preparations of the following concentrations: 720 nM, 240 nM, and 80 nM. Measurements of biolayer thickness were taken every 3 1/3 seconds for each sensor. Following the association step with the nucleic acids, the sensors were returned to binding buffer, and a change in thickness of the biolayer was measured for 15 minutes to determine dissociation rates. The Octet software was used for kinetics analysis.

### DNase Activity Assay

The recombinant his-tagged proteins isolated from E. coli (HctA, DdbA, and the his-tagged library) were incubated with plasmid DNA (pcDNA 6.2/N-EmGFP/GW/CAT) at various dilutions (1µg, 0.1µg, 0.01µg protein: 1µg plasmid DNA) in PBS. The recombinant 6xHis-tagged proteins isolated from Ctr (DdbA, Euo and Clover) were incubated with 1µg plasmid DNA. Recombinant protein was incubated with DNA for 5 minutes at 37°C, and then EDTA was immediately added to all samples to stop the reaction. Reaction mixes were then heated to 65°C for 10 minutes as a deactivation step. Reaction mixes were then incubated with 10 μg/mL proteinase K for 2 hours at 50°C. DNA was purified with phenol (isoamyl alcohol)-chloroform extraction. Gel electrophoresis was then performed on a 1.8% agarose gel.

### Genomic Isolation and Whole Genome Sequencing

Infected cells were rinsed with ice cold K36 scraped from the flask and stored on ice during the purification. The reticulate Bodies (RBs) and Elementary Bodies (EBs) were purified by sonication for EBs (48 hpi) and Dounce homogenization for RBs (18 hpi) before purification by density gradient (DG) centrifugation, essentially as described (Howard et al., 1974). Genomic DNA was isolated from both EBs and RBs purified from cells incubated at indicated temperatures in triplicate using Qiagen DNeasy (Qiagen) following the protocol provided. DNA samples isolated from infected cells were quantified and the libraries built and barcoded by the IBEST Genomics Resources Core at the University of Idaho. The libraries were sequenced using the Illumina Miseq platform at the IBEST Genomics Resources Core.

The sequencing reads were trimmed using bbduck (https://jgi.doe.gov/data-and-tools/bbtools/bb-tools-user-guide/bbduk-guide) aligned to the *Ctr* Bu 434 reference sequence (GCA_000068585.1 from ENA/EMBL) using bowtie2 (Langmead and Salzberg, 2012) and the resulting alignments were used by the iRep script (Brown et al., 2016) to calculate read bias across the chromosome. The iRep scripts generated a replication index corresponding to the bias of reads at the origin of replication as compared to the terminus of replication. iRep indexes were only reported if genome read coverage was at least 15 reads at each base location on the genome. Sequence data deposited with NCBI submission: SUB9741781.

## Results

### CTL0322 (DdbA) Is a DNA Binding Protein

**Figure 1:**
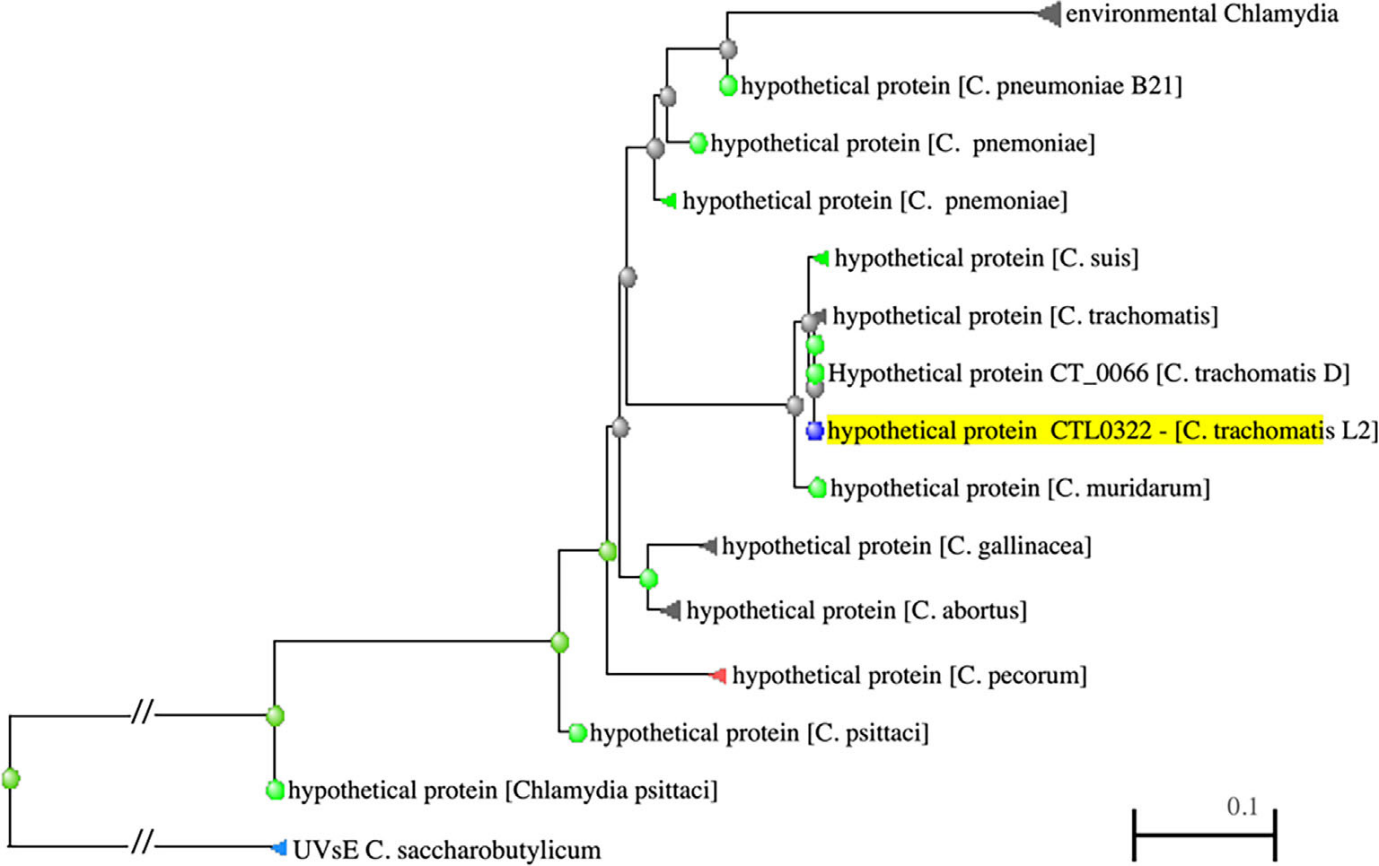
PSI-BLAST results showing distance tree of DdbA related proteins (highlighted). DdbA homologs are present in all the chlamydiaceae and are distantly related to an endonuclease in *Clostridium saccharobutylicum*.

The protein encoded by the *ddbA* gene is predicted to be a small, ~18kd, basic (pi = 9.95), cytoplasmic protein. A NCBI BLAST homology search revealed that DdbA is conserved throughout the chlamydial lineage; encoded in the genomes of all vertebrate *Chlamydia* pathogens as well as the environmental chlamydial parasites of unicellular organisms (**Figure 1**). The BLAST search revealed little insight into possible function as no conserved domains were identified. Interestingly, the only non-chlamydial hit was a putative UV damage endonuclease from *Clostridium saccharobutylicum* (**Figure 1**), although the homology was very low 36/131(27%) leading to low confidence in its predictive value. Additionally, the tool PredictProtein predicted that the product of *ddbA* contains multiple nucleic acid binding domains (Yachdav et al., 2014). The Nelson lab at the University of Indiana isolated a temperature sensitive Ctr L2 isolate that made significantly less EBs at the nonpermissive temperature of 40°C. Whole Genome Sequencing (WGS) revealed a mutation in the CTL0322 (ddbA) gene (Brothwell et al., 2014). Blast alignment of the DdbA protein demonstrates that this mutation is in a highly conserved region of the protein but that this particular amino acid charge, although rare, does occur in some variants (**Figure S1**).

**Figure 2:**
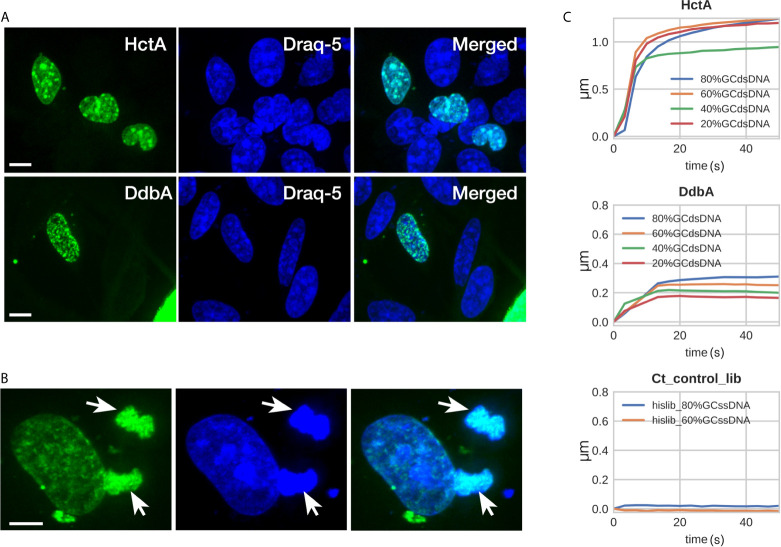
DdbA binds to DNA. Confocal images of DdbA ectopically expressed in HeLa cells **(A, B)** and Biolayer interferometry of purified DdbA with DNA **(C)** indicate an affinity for DNA. **(A)** HctA and DdbA were cloned and expressed with GFP-N terminal tags in HeLa cells. GFP signal is in green and DNA was stained with Draq5 (Blue) to highlight the nucleus. **(B)** Cell transfected with DdbA undergoing mitosis. Arrows indicate co-localization of the GFP signal with the mitotic chromosomes. Size bar = 10µm. **(C)** His-tagged DdbA and HctA were bound to a biolayer interferometry glass probe and binding measured using dsDNA ranging from 20%, 40%, 60% and 80% GC content. Both HctA and DdbA showed significant DNA binding with a maximal binding kinetics of 2.3 nM KD and 7.3 nM KD respectively. A his-tagged Ctr protein library (Ct_control_lib) was used as a negative control and showed no DNA binding (representative binding kinetics from one of three independent binding experiments).

Based on the predicted structure, it is likely that the DdbA protein is a chlamydial cytosolic protein and is not secreted from *Chlamydia*. However, with the thought that the protein’s subcellular localization may suggest function, a DdbA-GFP fusion was ectopically expressed in HeLa cells. The subcellular localization of the DdbA-GFP fusion was compared to an HctA-GFP fusion by confocal microscopy. Both fusion proteins were localized to the nucleus of the transfected cells during interphase (**Figure 2A**), while GFP alone was found throughout the cell (**Figure S2**). Additionally, DdbA-GFP co-localized with the mitotic chromosomes of dividing cells after the nuclear membrane was disrupted, suggesting that ectopically expressed DdbA binds to DNA and is not just nuclear localized (**Figure 2B**). This is consistent with the reported localization of the *C. trachomatis* serovar D homolog, CT066, as ectopic expression of this protein resulted in nuclear localization in both yeast and Hep2 cells (Sisko et al., 2006)

To directly test the hypothesis that DdbA is a nucleic acid binding protein, 6xHis tagged DdbA was expressed and purified from E. coli and the binding kinetics were measured using bio-layer interferometry (BLI) (ForteBio Octet). 6xHis tagged DdbA was bound to the tip of a glass probe and the association/dissociation between DdbA and DNA oligos were measured. DNA oligos of varying GC content were tested. HctA was used as a positive control and bound to DNA with little discernible preference for GC content. Although binding kinetics of the 40% GC oligo was lower than the others, no trend was evident (**Figure 2C**). The highest affinity of HctA binding was to the 60% GC oligo with a KD of 2.3 nM. DdbA bound DNA with a slight preference for higher GC content (**Figure 2C**). The KD for DdbA was calculated to be 7.3 nM for the 60% GC oligo. We also tested binding to both single stranded DNA and RNA. Both DdbA and HctA bound RNA and ssDNA but with reduced affinity as compared to dsDNA (**Figure S3**). As a negative control we generated a random chlamydial peptide 6xHis tagged library and tested it for DNA binding. In all cases the random peptide library did not bind DNA (**Figure 2C**).

**Figure 3:**
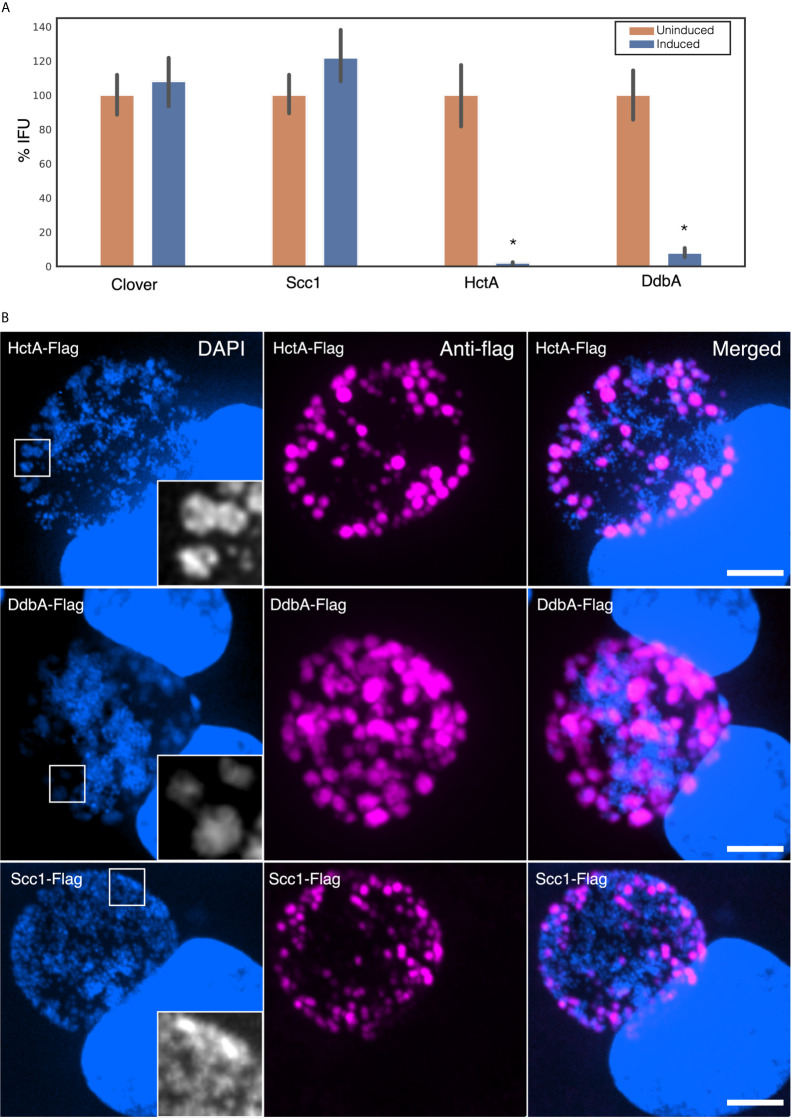
Ectopic expression of DdbA. **(A)** Cos-7 cells were infected with *Ctr* transformed with theophylline inducible expression plasmids controlling the production of HctA, DdbA, GFP (clover) or Scc1. Infected cells were induced for expression at 14 hpi and infectious progeny were isolated at 30 hpi. Progeny were quantified using an inclusion forming reinfection assay. Both HctA and DdbA induction resulted in a significant reduction in EB production (p < 0.01). While induction of Scc1 and GFP had no significant effect on EB production. **(B)** The morphological effects of ectopic expression was assessed by confocal microscopy. Cos-7 cells were infected with transformed Ctr expressing HctA, DdbA and Scc1, induced at 16 hpi, fixed at 24 hpi and stained with an anti-flag antibody to localize the expressed proteins. Additionally DNA morphology was assessed using DAPI staining. HctA expression resulted in a distinct change in the DAPI staining morphology, causing densely compacted regions of DNA in the RB. DdbA expression caused an increase in RB size but did not appreciatively change the compaction of the DNA. Scc1 expression had no effect on RB or DNA morphology. Size bar = 5µm. *p < 0.01.

Like HctA, DdbA is a small basic protein that localizes with DNA when ectopically expressed in mammalian cells, and is also under the regulatory control of the sRNA, IhtA (Grieshaber et al., 2012). We therefore compared the ectopic expression of DdbA to that of HctA in *Chlamydia*, both by IFU and confocal microscopy. *Ctr* were transformed with a plasmid encoding HctA, DdbA, Scc1, or GFP (clover) under the translational control of the E riboswitch (Topp and Gallivan, 2007). Cells infected with the transformed strains were treated with theophylline at 14 hpi and EBs were isolated at 30 hpi and quantified using a replating assay. HctA and DdbA expression dramatically reduced the number of infectious progeny produced after induction while ectopic expression of a non DNA binding chlamydial protein, Scc1, or GFP had no effect on progeny formation (**Figure 3A**). We also investigate the effects of ectopic expression on inclusion and *Chlamydia* morphology using confocal microscopy. HctA expressed in *E. coli* results in dramatic condensation of the chromosome, forming a densely compact DNA structure in the cell (Barry et al., 1992; Barry et al., 1993). When ectopically expressed in *Chlamydia* we see larger RBs with distinct densely compacted DNA regions (**Figure 3B**). Ectopic expression of DdbA also results in an increase in RB size but does not appear to compact the DNA (**Figure 3B**). Ectopic expression of Scc1 (a non DNA binding chlamydial protein) did not change the morphology of the RBs or EBs (**Figure 3B**). The evidence thus far suggests that *ddbA* encodes a DNA binding protein, but that this DNA binding protein does not function to compact DNA as does HctA.

### DdbA Has Nuclease Activity

**Figure 4:**
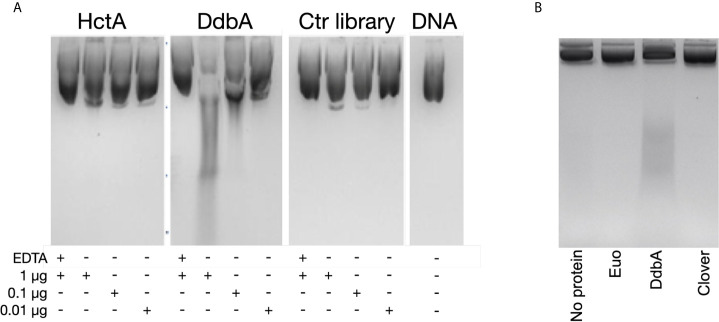
Purified DdbA displays nuclease activity. **(A)** The indicated concentrations of DdbA-6xHis, HctA-6xHis and a cocktail of purified proteins from a his tagged chlamydial protein expression library ectopically expressed and purified from *E coli* were incubated with plasmid DNA for 5 minutes at 37°C. Deproteinated, purified DNA was analyzed by agarose gel electrophoresis. EDTA was used as a control to demonstrate the nuclease activity was Mg++ dependent. HctA and the chlamydial library did not significantly change the migration of the DNA in the agarose gel. Purified DdbA cleaved DNA in a concentration dependent manner and was inhibited by EDTA. **(B)** Euo-6xHis, DdbA-6xHis and Clover-6xHis ectopically expressed in Ctr L2 were purified and incubated with 1µg of plasmid DNA for 5 minutes at 37°C. Deproteinated, purified DNA was analyzed by agarose gel electrophoresis. Only DNA incubated with DdbA-6xHis was cleaved.

Although protein homology database searches revealed very little about the function of DdbA, we have demonstrated it to be a DNA binding protein and the only suggestive hit from the homology search was an endonuclease (**Figure 1**). Interestingly, the BLI binding data show a slow negative slope for DdbA after fast binding to ssDNA and dsDNA with a slightly more negative slope for dsDNA, suggesting nuclease activity (**Figure S3**). However, the data suggest no degradation of the RNA probe suggesting the nuclease activity may be DNA specific (**Figure S3**). The HctA BLI data does not show this negative slope. Therefore, we tested DdbA for DNase activity against purified plasmid DNA. DdbA-6xHis, HctA-6xHis and a random 6xHis tagged Ctr peptide library were isolated from E.coli (**Figures S4A, B**). The purified proteins were incubated at varying concentrations with plasmid DNA. Incubation of DdbA with plasmid DNA caused an increase in fragmentation of the circular plasmid in a concentration dependent manner (**Figure 4A**). The nuclease activity of DdbA was also divalent cation dependent as EDTA was able to completely block this activity (**Figure 4A**). No similar activity was found in HctA or chlamydial protein library preparations purified from E.coli with the same affinity slurry and methods. This purification was repeated several times, with the same activity appearing each time.

We also performed these DNase experiments using 6xHis tagged proteins ectopically expressed and purified from C. trachomatis (**Figures S4C, D**). Equal amounts of purified DdbA, Euo and the fluorescent protein Clover were incubated with plasmid DNA (**Figure 4B**). Similar to the results above, only purified DdbA caused fragmentation of the plasmid DNA while incubation with purified Euo or Clover did not.

### DdbA Is Involved in Proper EB Formation

**Figure 5:**
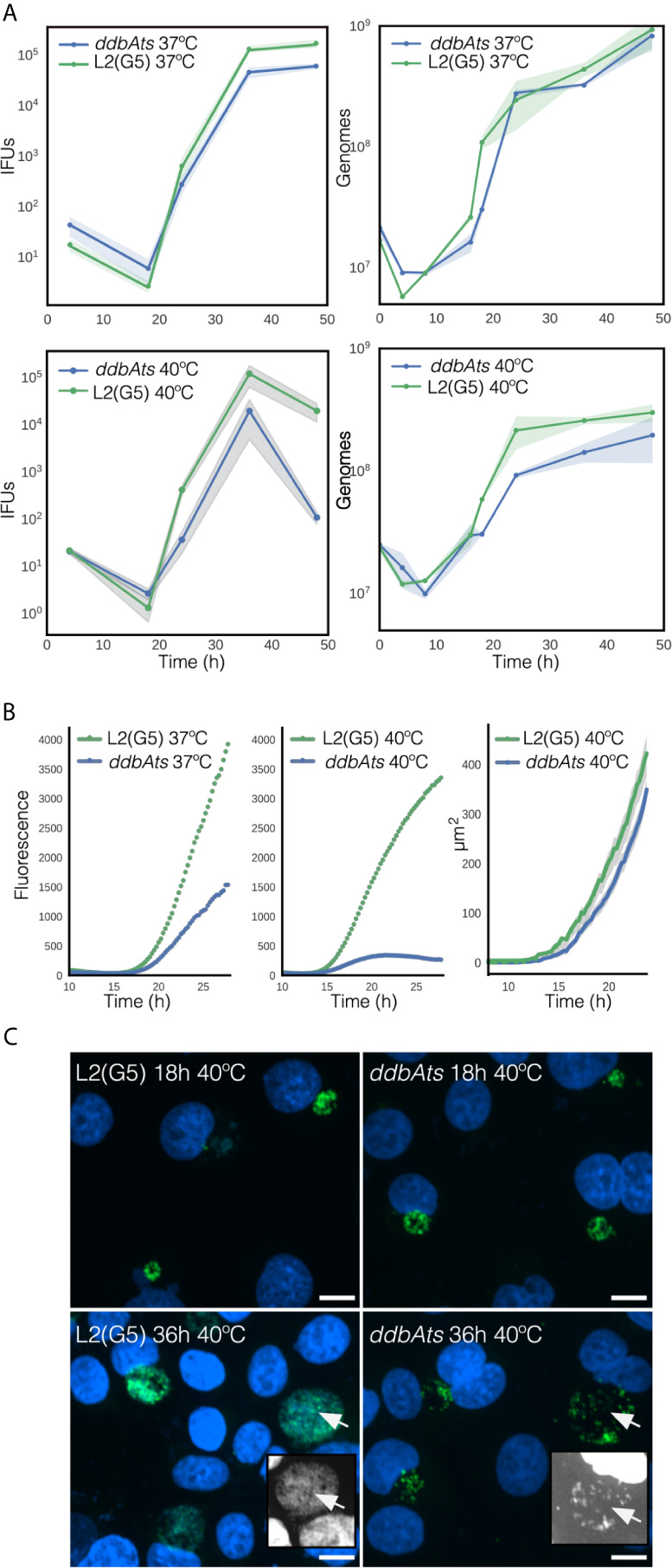
Growth and infectivity characteristics of the *ddbAts* mutant. **(A)** The ddbAts chlamydial mutant was grown at 37°C or 40°C and production of infectious progeny (IFUs) and genomic copy number, were compared to the isogenic strain L2(G5). There was little difference in growth and progeny production between L2(G5) and the ddbAts mutant at 37°C but at 40°C there was a dramatic difference in IFU counts late in the infection. **(B)** Live cell analysis of ddbAts late gene expression and comparison of inclusion size. ddbAts and L2(G5) were transformed with a plasmid expressing the fluorescent protein Clover driven by the hctA promoter and imaged every 15 minutes for 30 hours at either 37°C or 40°C. The size of the chlamydial inclusions was determined by measurement of inclusions from the microscopy images at the nonpermissive temperature of 40°C. The cloud represents 95% confidence intervals and n > 50 inclusions were measured per experiment. **(C)** The ddbAts defective phenotype is only observable during EB production. Confocal images of ddbAts growth showed that the inclusions are nearly identical to L2(G5) at 18 hpi but that at 36 hpi the ddbAts mutant inclusion contains less chlamydial organisms. L2(G5) and ddbAts mutants grown at 40°C for 18 and 36 hours were fixed and stained with DAPI to label the DNA (blue) and an anti-MOMP antibody to label Chlamydia (green). Insert pictures highlight the DAPI channel to better visualize the EBs. Size bar = 10µm.

We suspect that *ddbA* is an essential gene as no frameshift or nonsense mutations were isolated in a saturation mutagenesis screen performed in the Valdivia lab at Duke University (Nguyen and Valdivia, 2011). However, as mentioned above the Nelson lab at the University of Indiana isolated a temperature sensitive *Ctr* L2 isolate that made significantly less EBs at the nonpermissive temperature of 40°C. A nearly isogenic strain (G5) was isolated from a backcross of *ddbA*ts to a second temperature sensitive isolate containing the *ddbA*ts mutation (Brothwell et al., 2014). All progeny that contained the *ddbA*ts mutation retained the temperature sensitive phenotype while all others, one of which was named G5, regained wt growth. Thus, the presence of the *ddbA*ts mutation was required for the temperature sensitive phenotype. Both the *ddbA*ts and the G5 strain were kindly provided to us by Dr. Nelson. To determine the stage of the developmental cycle disrupted by the elevated temperature, we determined the growth rate (genomic copy number) and production of EBs (infectious progeny) over the entire developmental cycle for both *ddbA*ts and *Ctr* L2(G5). Both growth and production of infectious progeny were nearly indistinguishable between the isolates when incubated at the permissive temperature of 37°C (**Figures 5A, B**). However, when grown at 40°C, the *ddbA*ts mutant showed significant differences late during infection. Genomic copy number was similar to G5 throughout the infection however the number of infectious progeny was dramatically lower overall even though production of EBs was initiated at a similar time (**Figure 5B**).

To follow the cycle more closely, we developed a reporter system to track development in real time. *Ctr* L2(G5) and *ddbAts* strains were both transformed with a plasmid containing the promoter and first ten amino acids of the late gene *hctA* fused to a fluorescent protein (Chiarelli et al., 2020) This region of the *hctA* gene contains both the promoter and the regulatory region targeted by the sRNA, IhtA (Grieshaber et al., 2012). When transformed into *Ctr* L2(G5), the GFP signal could be detected at ~15 hours post infection and showed an increase in expression that mimicked the production of EBs (**Figure 5B**). At 37°C, live cell imaging of the *ddbAts* mutant strain demonstrated that expression of the late gene reporter was detected at the same time as G5, but showed a slower overall expression rate over time. However, growth at the nonpermissive temperature showed a dramatic difference in the late gene reporter expression compared to 37°C (**Figure 5B**). At 40°C, detection of late gene expression in both strains was shifted a few hours earlier and was detectable ~13.5 hours post infection. Contrary to wild type G5 L2 late gene expression which increased logarithmically after first detection, expression of the reporter in the *ddbAts* strain increased only moderately and was followed by a decrease in expression ~20 hour post infection (**Figure 5B**). To determine if inclusion size was affected at the non-permissive temperature, inclusions were measured using live cell imaging and the mutant compared to L2(G5). Inclusion size between G5 and the *ddbA*ts mutant was quite similar. Both strains displayed dramatic inclusion expansion at ~14 hours post infection (hpi) that correlated with expression of the late gene reporter (**Figure 5B**). However, unlike late gene expression, inclusion expansion was only moderately affected in the mutant at 40°C with expansion continuing past 24 hours (**Figure 5B**).

As inclusion size was comparable between G5 and the mutant but IFU counts and late gene expression differed dramatically, inclusion morphology of the strains incubated at both the permissive temperature (37°C, **Figure S5**) and the non-permissive temperature (40°C, **Figure 5C**) were investigated by confocal microscopy. At 37°C the inclusions of both strains looked similar in morphology and size at 18 hpi and 36 hpi (**Figure S5**). The inclusions at the 18 hour time point for both strains grown at 40°C were also similar in size and appeared to contain numerous RB cell forms (**Figure 5C**). However, by 36 hpi, the inclusions in the ddbAts infected cells incubated at 40°C were dramatically different in appearance. Although the inclusions were similar in size, they appeared to have fewer organisms in the inclusion lumen leading us to suspect that the ddbAts strain was making fewer EB cell forms or that these cell forms lysed after formation (**Figure 5C**).

### Maintenance of EB Infectivity

**Figure 6:**
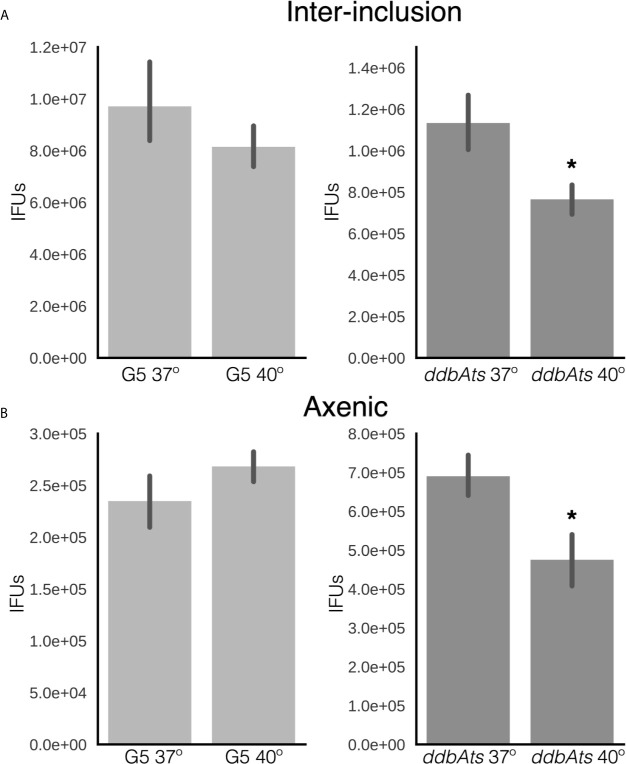
EBs produced by *ddbAts* mutant cells have a defect in maintenance of EB infectivity at the non-permissive temperature. **(A)** The ability of EBs to maintain infectivity after formation was determined by treating infected cells grown at 37°C with penicillin at 24 hpi and then shifting the temperature to 40°C. The premade EBs were harvested at 48 hours and maintenance of infectivity measured by IFU assay at 37°C. **(B)** Maintenance of infectivity was also measured using an axenic growth media. EBs from G5 or the ddbAts mutant were incubated in CIP-1 axenic growth media at 37°C or 40°C for 24 hours before measuring infectivity by an IFU assay. *p < 0.01.

We reported previously that the chlamydial EB actively maintains infectivity after differentiation, both within the inclusion and in a synthetic growth medium containing an energy source (Grieshaber et al., 2018). Therefore, we asked whether the *ddbAts* mutant impacted long term infectivity of preformed EBs. To test the viability of the *ddbAts* EBs in the interinclusion space, continued EB production after the initial rounds of differentiation was blocked by treatment with penicillin (Grieshaber et al., 2018). *Ctr* does not use peptidoglycan as a structural sacculus and EBs do not contain a peptidoglycan cell wall. Instead, peptidoglycan aids cell septation by forming a ring at the cleavage furrow (Liechti et al., 2013). Therefore, *Ctr* treated with penicillin cease to divide and do not produce new EBs (Grieshaber et al., 2006; Chiarelli et al., 2020). The mechanism linking the completion of cell division and EB development is not currently known but it has been reported in multiple studies that addition of penicillin blocks EB formation (Matsumoto and Manire, 1970; Lambden et al., 2004; Grieshaber et al., 2006; Skilton et al., 2007). Recently published models of EB development also proposed cell division as being linked to EB development (Lee et al., 2016; Chiarelli et al., 2020). To test EB viability, cells were infected with either L2(G5) or the *ddbAts* mutant for 36 hours at 37°C, at which point further EB production was inhibited by addition of penicillin (Pen) and cultures were either shifted to 40°C or left at 37°C. Treated bacteria were harvested at 60 hpi (24 h Pen). EB infectivity was measured by a conventional IFU assay (**Figure 6A**). There was no statistical difference in EB viability between 40°C and 37°C for L2(G5) EBs. In the *ddbAts* mutants however, there was a significant reduction in viability in the 40°C shifted EBs (**Figure 6A**).

Viability of EBs incubated in axenic media was also tested. Equal numbers of purified *ddbA*ts or L2(G5) were incubated for 18 hours at 37°C or 40°C in Cip-1 media (Omsland et al., 2011; Grieshaber et al., 2018). EB infectivity was measured using re-infection *via* a traditional IFU assay. No significant difference in maintenance of EB infectivity was detected for *Ctr* L2(G5) between the two temperatures. However, there was a significant loss in infectivity of the *ddbAts* EBs incubated at 40°C (**Figure 6B**). These data suggest DdbA may be important in both the production and maintenance of infectivity of the EB cell form. However, the decrease in EB viability is slight compared to the overall decrease in EB production during development. We hypothesize that the function of DdbA could overlap between the two cell states i.e. EB committed cells and infectious EBs. EB production is likely to be complicated as we have shown that EB maturation, from cells committed to differentiation to mature infective EBs, takes ~8 hours (Chiarelli et al., 2020).

### Chromosomal Replication Index

**Figure 7:**
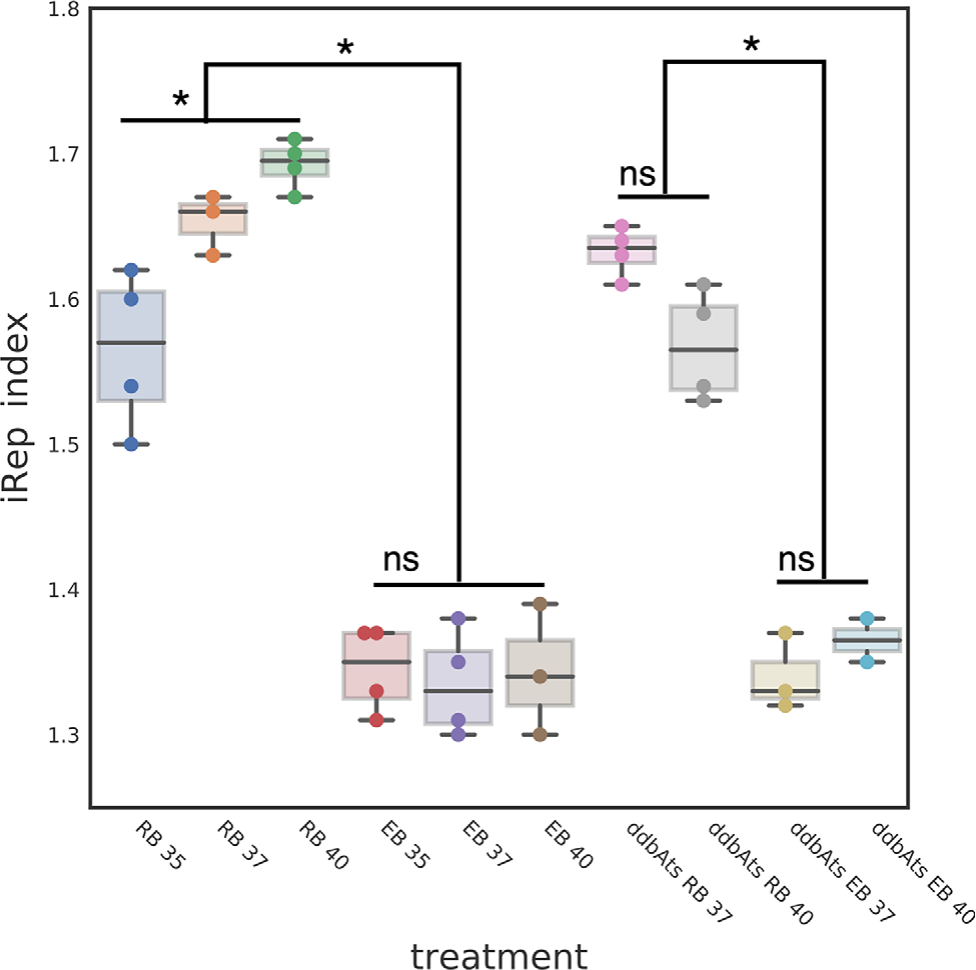
DdbA is not involved in the RB to EB replication check point. The EB cell type index of replication suggests a DNA replication initiation checkpoint is a component of the chlamydial developmental cycle. The index of replication was determined using the iRep script which calculates the sequence read bias at the origin of replication as compared to the terminus. The iRep index for L2 wt RBs was temperature dependent; RBs grown at 35°C had an iRep index of 1.57, at 37°C an index of 1.66 and at 40°C an index of 1.70. These differences were statistically significant with p < 0.001. The iRep index for L2 wt EBs was much lower and showed no temperature dependent changes (1.35, 1.33, and 1.34 respectively). The ddbAts mutant showed the same significant difference in the iRep between the RB cell type and EB cell types (1.63 and 1.33). This difference was also evident at the nonpermissive temperature (RB = 1.56 and EB = 1.36). *p < 0.001. NS, not significant.

Our data suggests that DdbA interacts with DNA and contains nuclease activity, is specific to and conserved throughout the chlamydial lineage, and acts during the critical transition from the replicating RB to the replication quiescent EB cell form. Due to the lack of homologies outside of the chlamydial family we speculate that DdbA acts in a chlamydial specific pathway. The condensation of the *Chlamydia* chromosome is unique among bacteria and likely introduces unique stresses on the chromosome. This would be especially true if the chromosome is being actively transcribed and replicated during condensation, the step thought to halt EB chromosome replication. This unique biology may necessitate novel proteins with nuclease activity. To determine if the EB nucleoid compaction occurred concurrent with active DNA replication, we employed WGS and iRep analysis. The iRep WGS analysis tool chain was developed to determine replication rates from genomic samples by calculating the sequence read coverage bias between the origin of replication and replication terminus (Brown et al., 2016). If compaction occurred concomitantly with replication, the EB would contain a similar number of partially replicated genomes as the RB cell form, indicated by a similar iRep index. *Ctr* L2 wt was grown at three different temperatures to generate RBs replicating at different rates. Cells were infected with *chlamydia* and grown at 35°C, 37°C or 40°C and RB cell forms were purified at 18 hpi while EB cell forms were harvested at 48 hpi. The genomic DNA was subjected to WGS and an index of replication was calculated using the iRep scripts. (Brown et al., 2016). As expected the replication index for RBs increased with temperature (1.57 ± 0.03@35°C, 1.66 ± 0.02@37°C and 1.70 ± 0.02@40°C). Surprisingly, the replication index for the EBs was significantly lower than in the RBs and did not vary with temperature (1.35 ± 0.02@35°C, 1.33 ± 0.02@37°C and 1.34 ± 0.03@40°C) (**Figure 7**). The decrease in the iRep index and the lack of a temperature response indicates that initiation of DNA replication is actively inhibited during the RB to EB conversion and that the replication forks proceed to completion prior to chromosome condensation. The uncoupling of DNA replication initiation from DNA replication is reminiscent of cell cycle checkpoints in eukaryotic cells and the bacterium *Caulobacter crescentus* (Ausmees and Wagner, 2003; Gorbatyuk and Marczynski, 2005).

To determine if DdbA is implicated in this checkpoint, the replication index of the ddbAts mutant was measured at the permissive 37°C and nonpermissive 40°C temperatures for both RBs and EBs. The replication index for the ddbAts mutant RBs grown at 37°C was similar to wt (1.63 ± 0.02) but was decreased at 40°C (1.56 ± 0.02). The iRep index of the ddbAts EBs mimicked wt (**Figure 7**). The iRep index was again significantly lower in the EBs compared to the RBs and was temperature independent (1.33 ± 0.02 and 1.36 ± 0.01 respectively) (**Figure 7**. Taken together, this data is suggestive of a RB/EB cell cycle control checkpoint, but that ddbA does not contribute to this regulation.

## Discussion

The chromosomes of bacteria are highly structured and compacted in order to fit inside of the cell. This structure must be organized to facilitate replication, transcription and chromosome segregation. Most bacteria encode small, and usually basic proteins, called nucleoid associated proteins (NAPs) (Kasinsky et al., 2001). These proteins typically bind somewhat nonspecifically and can either wrap DNA [*e.g.* HU (Dillon and Dorman, 2010)], bend DNA [*e.g.* IHF (Stella et al., 2010) and Fis (Guo and Adhya, 2007)], or bridge distal pieces of DNA (*e.g.* H-NS (Dame et al., 2006)). The chlamydial genome contains only one conserved NAP; a single gene for a protein that is homologous to the HU/IHF family of heterodimeric NAPs. However, the unique biology of Chlamydia creates additional requirements for DNA packaging. Transition from the RB form to the EB form requires many coordinated changes in the cell. The cells become reduced in size, over 85% in volume, the chromosome is compacted extensively, translation and replication are halted, and proteins in the outer membrane become disulfide cross linked (Abdelrahman and Belland, 2005). Chromosome compaction is accomplished in part by two chlamydial specific NAPs, HctA and HctB, which have homology to linker histones in archaea and eukaryotic cells (Hackstadt et al., 1991; Tao et al., 1991; Perara et al., 1992; Brickman et al., 1993).

Our data suggest that the product of *ddbA* is involved in the transition of the RB cell type to the EB form. We had previously shown that DdbA expression is regulated by the sRNA IhtA, placing it in the same regulon as the chlamydial histone protein HctA and suggesting a role in the formation of the compacted EB nucleoid (Grieshaber et al., 2012). We show that like HctA, DdbA is a DNA binding protein, however, unlike HctA, it does not contribute directly to chromosome compaction. G5 and ddbAts growth and development differ significantly when grown at the non-permissive temperature of 40°C. The seemingly contradictory observed delay between HctA expression decrease (~20 hpi) and the decrease in IFUs (>36 hpi) agrees well with our published observation that there is a 10-12 hour delay between HctA expression and EB infectivity (Chiarelli et al., 2020).

Our data also indicates that DdbA potentially encodes nuclease activity. A caveat to the DNase assay performed with protein isolated from both *E. coli* and *Ctr* is that the proteins were not isolated to purity (**Figure S4**) and therefore a nuclease may be present as a result of co-precipitating contamination during the protein purification. This concern is somewhat allayed as no similar activity was found in the HctA and chlamydial protein library controls (isolated from *E. coli*), or Euo and Clover preparations (isolated from *Ctr*). If a co-purifying protein is present then it is likely biologically significant as it would have been co-purified with DdbA isolated from both *E. coli* and *Chlamydia*. Additionally, we purified ectopically expressed DdbA containing a flag-tag from *Ctr* and also observed DNase activity; however we switched to the 6xHis tag as we were unable to purify the flag-tagged controls (data not shown). Taken together, we believe it is likely that DdbA either has nuclease activity itself or is co-purifying with a protein with biologically significant activity.

We do not yet know the functional role of DdbA in EB formation and maintenance of infectivity but the need for specialized nucleases to relieve strain is an intriguing possibility as histone mediated EB nucleoid formation would lead to DNA winding stress as well as other potential topological stresses in the DNA. Bacterial DNA replication initiates from a single site on the chromosome and proceeds bidirectionally (Gros et al., 2002; Brown et al., 2016). To achieve high replication rates, bacteria initiate the next chromosomal replication before the first replication is complete leading to more copies of the DNA content near the origin of replication as compared to replication terminus. Our iRep data confirm that chlamydial RBs replicate similarly. The data show that RBs replicating faster at 40°C have a higher iRep index while RBs replicating slower at 35°C show a lower iRep index and RBs at 37°C show an intermediate iRep index. The chlamydial EBs however have an iRep index significantly lower than the RB cell forms and the iRep index is independent of temperature suggesting that initiation of DNA replication is blocked but the replication forks continue during EB formation. This is reminiscent of licensing schemes used in eukaryotic and some prokaryotic cell cycle control systems. The bacteria *Caulobacter crescentus* is a classical model to study the regulation of the bacterial cell cycle. It divides asymmetrically, resulting in a stalked cell that immediately enters S phase and a swarmer cell that arrests in G1 phase until it differentiates into a stalked cell. *C. crescentus* chromosomal replication is tightly regulated and occurs only in stalked cells and only once per cell cycle (O’Neill and Bender, 1989; Ozaki et al., 2014). Our iRep data is suggestive of a cell cycle control system in *Chlamydia* that allows replication in the RB cell type but suppresses DNA replication initiation upon RB differentiation to the EB cell. One of the mechanisms to protect DNA during packaging is to separate chromosome condensation from DNA replication. Our iRep data suggest that chlamydia does just that during EB formation. The iRep index for all EBs was significantly lower and not influenced by RB replication rates strongly suggesting that upon commitment to the EB cell form, initiation of DNA replication is blocked thereby separating DNA replication from chromosome compaction. This replication block was true for both the wt and *ddbAts* mutant suggesting that DdbA is not involved in the regulation of this process but more likely functions at the stage of EB DNA remodeling.

The unique biology of the chlamydial nucleoid likely drives the need for novel DNA binding proteins to facilitate the transition from a replicating, transcriptionally accessible chromosome in the RB to a highly compacted nonreplicating and transcriptionally quite chromosome in the EB cells form. Although the exact function of DdbA is currently not elucidated, our data demonstrate that it likely functions in this critical pathway. Additionally the iRep index data suggests that during the transition from a replicating RB chromosomes to the compacted EB chromosome replication initiation is suppressed while replication fork progression continues. This is suggestive of a replication licensing system potentially analogous to that of other bacteria with developmental cell forms such as *C. crescentus* (Marczynski and Shapiro, 2002; Gorbatyuk and Marczynski, 2005; Frandi and Collier, 2019). Elucidation of the control of DNA replication and details involved in DNA packaging will likely be an important component in understanding EB development.

## Data Availability Statement

The original contributions presented in the study are publicly available in NCBI using accession number PRJNA733157.

## Author Contributions

NG and SG designed and performed experiments, analyzed data, and wrote the manuscript. JR, AO, ST, MD, and CA performed experiments and analyzed data. All authors contributed to the article and approved the submitted version.

## Conflict of Interest

The authors declare that the research was conducted in the absence of any commercial or financial relationships that could be construed as a potential conflict of interest.

## Publisher’s Note

All claims expressed in this article are solely those of the authors and do not necessarily represent those of their affiliated organizations, or those of the publisher, the editors and the reviewers. Any product that may be evaluated in this article, or claim that may be made by its manufacturer, is not guaranteed or endorsed by the publisher.

## Supplementary Material

Supplementary Figure 1 Blast results showing the first 100 amino acids of DdbA. Overall homology is limited to the chlamydial family of organisms and ranges from ~40% to 100% homology. The red coloring shows conservation levels across all organisms with brighter red corresponding to higher levels of variation. Highlighted in green is the position of the M to I mutation in the ddbAts mutant (Brothwell et al., 2014). This mutation is in a highly conserved region. M to I and M to L variants appear to be rare but do occur across different species.

Supplementary Figure 2 Ectopically expressed GFP localized throughout the cell. Confocal images of GFP ectopically expressed in HeLa cells. GFP signal is in green and the nucleus is blue. Size bar = 10µm

Supplementary Figure 3 DdbA binds ssDNA and RNA. His-tagged DdbA and HctA were bound to a biolayer interferometry glass probe and binding measured using dsDNA, ssDNA or RNA with a 40% GC content. Both DdbA and HctA bound RNA and ssDNA but with reduced affinity as compared to dsDNA.

Supplementary Figure 4 Analysis of protein purification from *E.coli* and *Chlamydia trachomatis (Ctr)*. **(A)** Coomassie stain of 6xHis tagged DdbA, HctA and Ctr library purified from E. coli and separated on a 12% SDS-PAGE gel. The position of each protein is indicated. **(B)** Western analysis of 6xHis tagged DdbA and HctA purified from E. coli. **(C)** SYPRO Orange staining of 6xHis tagged DdbA, Clover and Euo purified from Ctr. **(D)** Western analysis of 6xHis tagged DdbA, Clover and Euo purified from Ctr.

Supplementary Figure 5 Inclusion phenotype of ddbAts and L2(G5) grown at the permissive temperature. Confocal images of ddbAts growth at 37°C indicate that the inclusions are nearly identical to L2(G5) at 18 hpi and 36 hpi. L2(G5) and ddbAts mutants grown at 37°C for 18 and 36 hours were fixed and stained with DAPI to label the DNA (blue) and an anti-MOMP antibody to label Chlamydia (green). Size bar = 10µm.
