# Supplementary figures and images for: The sRNA Regulated Protein DdbA Is Involved in Development and Maintenance of the Chlamydia trachomatis EB Cell Form
Source: Front Cell Infect Microbiol. 2021 Jul 23;11:692224. doi: 10.3389/fcimb.2021.692224 (PMC8343073; doi:10.3389/fcimb.2021.692224)

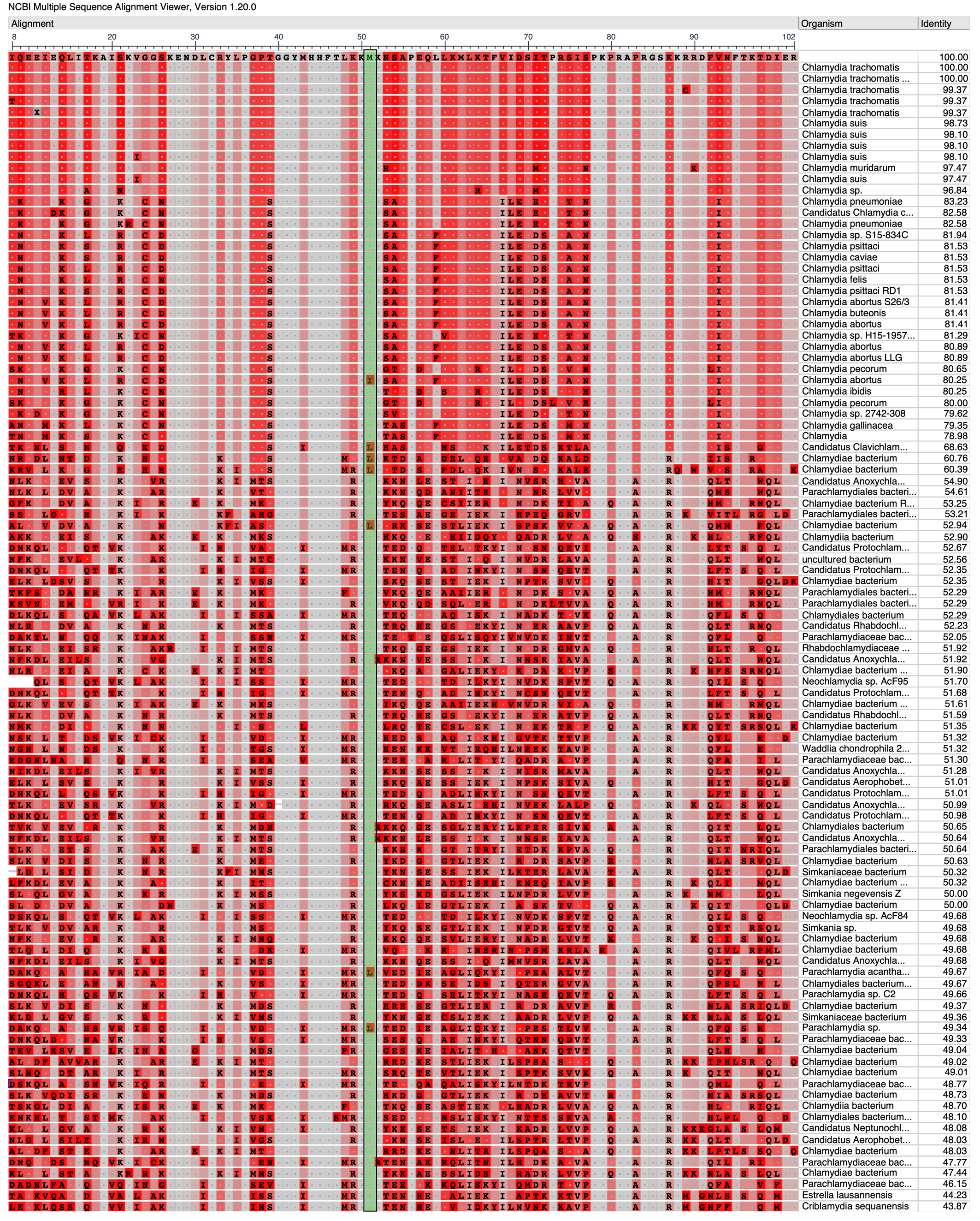

Supplement: Supplementary Figure 1 — Blast results showing the first 100 amino acids of DdbA. Overall homology is limited to the chlamydial family of organisms and ranges from ~40% to 100% homology. The red coloring shows conservation levels across all organisms with brighter red corresponding to higher levels of variation. Highlighted in green is the position of the M to I mutation in the ddbAts mutant (Brothwell et al., 2014). This mutation is in a highly conserved region. M to I and M to L variants appear to be rare but do occur across different species. [file Image_1.tif]

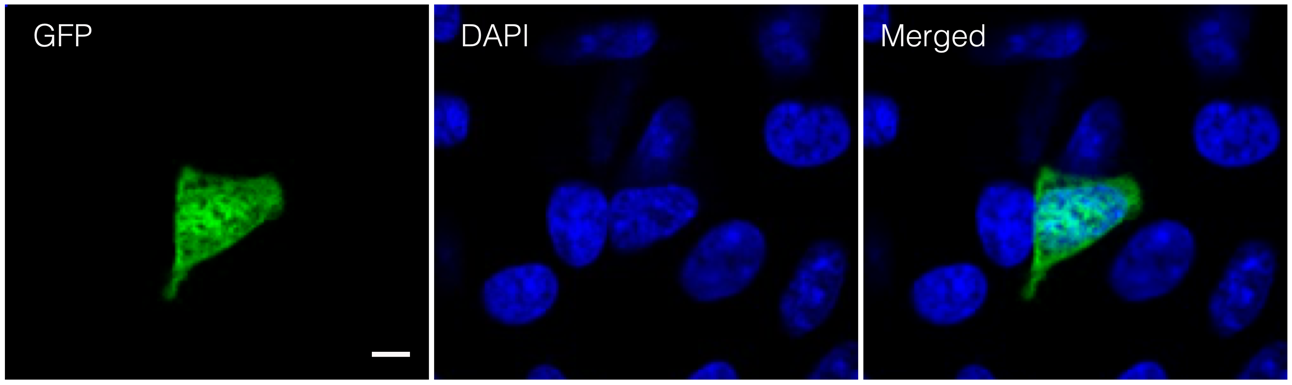

Supplement: Supplementary Figure 2 — Ectopically expressed GFP localized throughout the cell. Confocal images of GFP ectopically expressed in HeLa cells. GFP signal is in green and the nucleus is blue. Size bar = 10µm [file Image_2.tif]

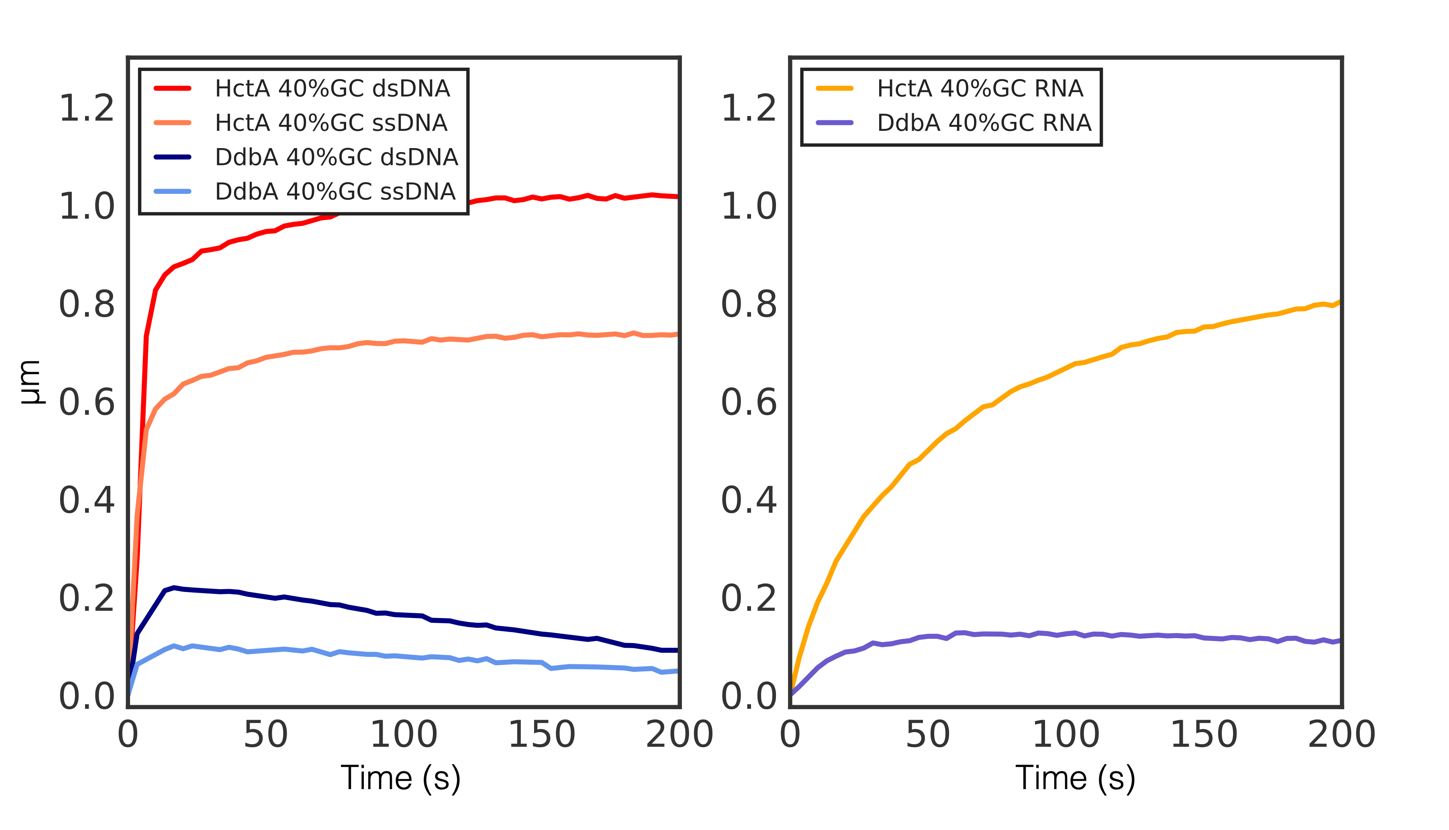

Supplement: Supplementary Figure 3 — DdbA binds ssDNA and RNA. His-tagged DdbA and HctA were bound to a biolayer interferometry glass probe and binding measured using dsDNA, ssDNA or RNA with a 40% GC content. Both DdbA and HctA bound RNA and ssDNA but with reduced affinity as compared to dsDNA. [file Image_3.tif]

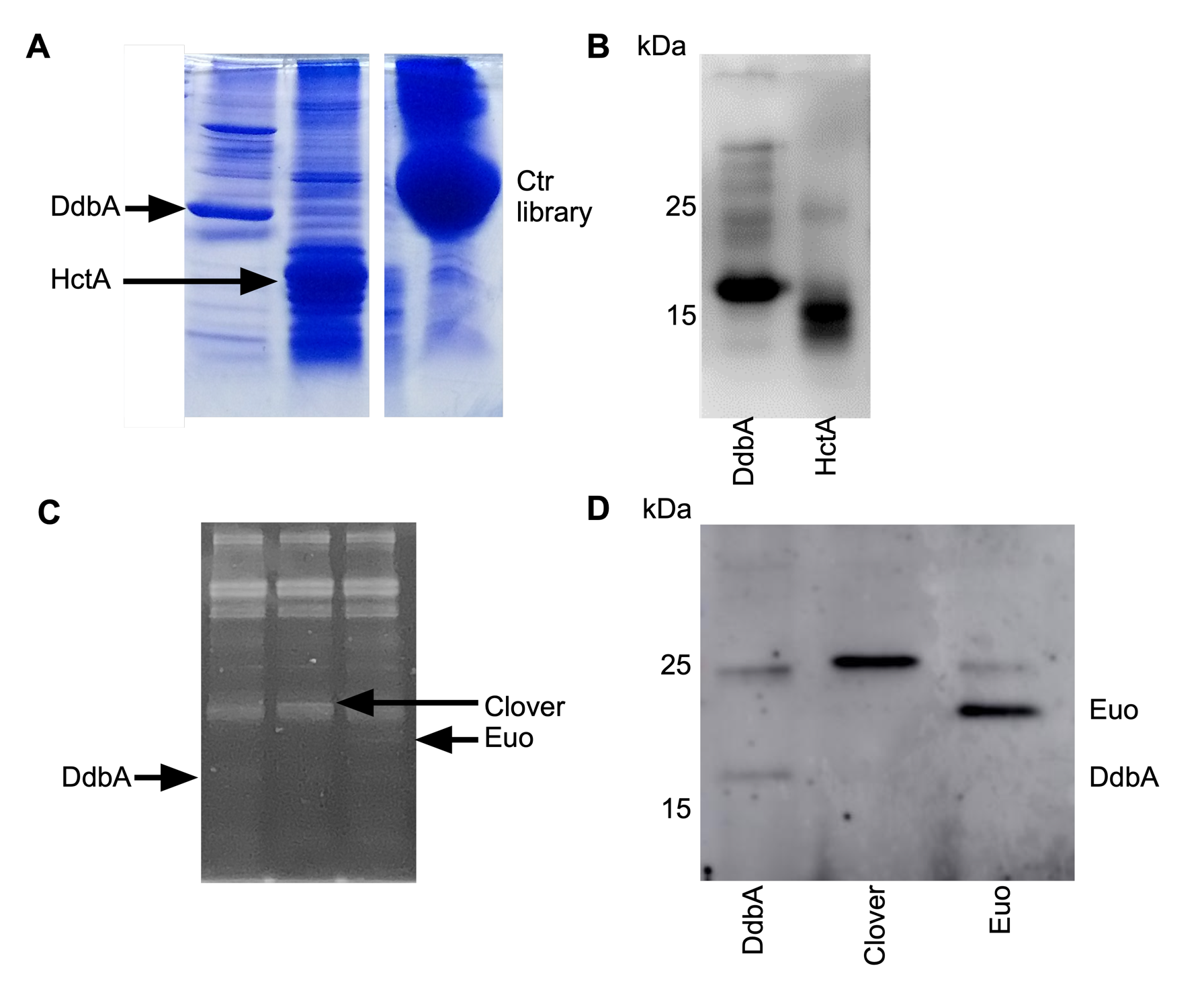

Supplement: Supplementary Figure 4 — Analysis of protein purification from E.coli and Chlamydia trachomatis (Ctr). (A) Coomassie stain of 6xHis tagged DdbA, HctA and Ctr library purified from E. coli and separated on a 12% SDS-PAGE gel. The position of each protein is indicated. (B) Western analysis of 6xHis tagged DdbA and HctA purified from E. coli. (C) SYPRO Orange staining of 6xHis tagged DdbA, Clover and Euo purified from Ctr. (D) Western analysis of 6xHis tagged DdbA, Clover and Euo purified from Ctr. [file Image_4.tif]

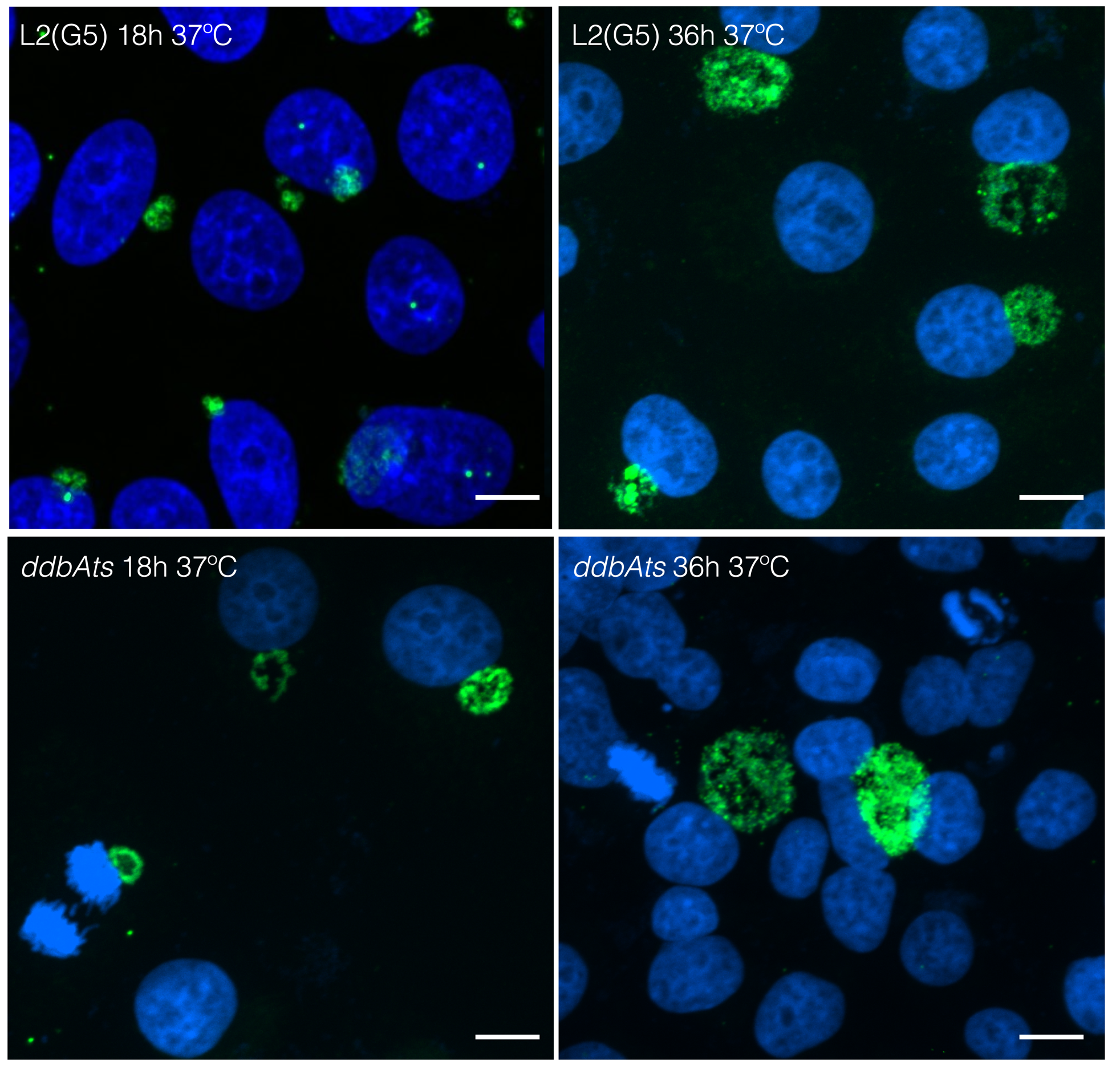

Supplement: Supplementary Figure 5 — Inclusion phenotype of ddbAts and L2(G5) grown at the permissive temperature. Confocal images of ddbAts growth at 37°C indicate that the inclusions are nearly identical to L2(G5) at 18 hpi and 36 hpi. L2(G5) and ddbAts mutants grown at 37°C for 18 and 36 hours were fixed and stained with DAPI to label the DNA (blue) and an anti-MOMP antibody to label Chlamydia (green). Size bar = 10µm. [file Image_5.tif]
